# Detection of Breathing Movements of Preterm Neonates by Recording Their Abdominal Movements with a Time-of-Flight Camera

**DOI:** 10.3390/pharmaceutics13050721

**Published:** 2021-05-14

**Authors:** Felix C. Wiegandt, David Biegger, Jacob F. Fast, Grzegorz Matusiak, Jan Mazela, Tobias Ortmaier, Theodor Doll, Andreas Dietzel, Bettina Bohnhorst, Gerhard Pohlmann

**Affiliations:** 1 Division of Translational Biomedical Engineering, Fraunhofer Institute for Toxicology and Experimental Medicine ITEM, 30625 Hannover, Germany; davidbiegger@gmail.com (D.B.); theodor.doll@item.fraunhofer.de (T.D.); 2 Institute of Mechatronic Systems, Leibniz Universität Hannover, 30823 Garbsen, Germany; jacob.fast@imes.uni-hannover.de (J.F.F.); tobias.ortmaier@imes.uni-hannover.de (T.O.); 3 Department of Phoniatrics and Pediatric Audiology, Hannover Medical School, 30625 Hannover, Germany; 4 Division of Infectious Diseases, Department of Neonatology, Poznan University of Medical Sciences, 61-701 Poznan, Poland; matusiak.grzegorz@gmail.com (G.M.); janco@pol-med.com.pl (J.M.); 5 Department of Otorhinolaryngology, Hannover Medical School, 30625 Hannover, Germany; 6 Institute of Microtechnology, Technische Universität Braunschweig, 38124 Braunschweig, Germany; a.dietzel@tu-braunschweig.de; 7 Department of Pediatric Pulmonology, Allergology and Neonatology, Hannover Medical School, 30625 Hannover, Germany; bohnhorst.bettina@mh-hannover.de

**Keywords:** abdominal movement, time-of-flight camera, preterm neonate, optical detection of breathing movements

## Abstract

In order to deliver an aerosolized drug in a breath-triggered manner, the initiation of the patient’s inspiration needs to be detected. The best-known systems monitoring breathing patterns are based on flow sensors. However, due to their large dead space volume, flow sensors are not advisable for monitoring the breathing of (preterm) neonates. Newly-developed respiratory sensors, especially when contact-based (invasive), can be tested on (preterm) neonates only with great effort due to clinical and ethical hurdles. Therefore, a physiological model is highly desirable to validate these sensors. For developing such a system, abdominal movement data of (preterm) neonates are required. We recorded time sequences of five preterm neonates’ abdominal movements with a time-of-flight camera and successfully extracted various breathing patterns and respiratory parameters. Several characteristic breathing patterns, such as forced breathing, sighing, apnea and crying, were identified from the movement data. Respiratory parameters, such as duration of inspiration and expiration, as well as respiratory rate and breathing movement over time, were also extracted. This work demonstrated that respiratory parameters of preterm neonates can be determined without contact. Therefore, such a system can be used for breathing detection to provide a trigger signal for breath-triggered drug release systems. Furthermore, based on the recorded data, a physiological abdominal movement model of preterm neonates can now be developed.

## 1. Introduction

Preterm neonates’ respiratory parameters differ greatly from those of adults. In contrast to adults, who have a tidal volume of approximately 500 mL, a respiratory rate of 15 breaths per minute and an inhalation-exhalation ratio (I:E) of 1:1, preterm neonates have a significantly lower tidal volume of 4–8 mL/kg, a higher respiratory rate of up to 60/min and an I:E of up to 1:3, depending on gestational age [1,2,3]. Therefore, the determination of respiratory parameters in preterm neonates is very challenging, and not every monitoring system is suitable for this task. However, the respiratory cycle must be known with high precision for the safe and effective operation of breath-triggered drug release systems for neonates. One of the primary drawbacks of inhalation therapy using a continuous drug delivery system is the substance loss during exhalation [4,5]. This loss mainly depends on the patient’s I:E. In addition, pharmaceutical aerosols can only reach the alveoli during the first half of the inspiratory phase [6], which may result in an increased aerosol loss of up to 90% in preterm neonates without breath-triggered administration. This high loss is in accordance with previous reports of low deposition in infants [7,8,9,10], in some cases of less than 1% of the nominal dose [11]. Therefore, breath-triggered drug release is highly desirable. It allows for the patient-specific delivery of pharmaceutical aerosols and thus has the following advantages over continuous drug release:

1. The previously mentioned loss during exhalation is theoretically reduced to zero, as the pharmaceutical aerosol is only released during inhalation. This leads to higher drug utilization and substantial cost savings [12,13,14].
2. Different lung regions can be targeted, as the pharmaceutical aerosol can be released as a bolus at different, pre-defined instants during the inhalation phase. A release at the beginning of inhalation mainly targets peripheral lung regions, while a release towards the end of inhalation mainly targets central lung regions. This also results in a more time-efficient treatment and a reduced distribution of the drug in the body, which reduces side effects [15,16,17,18,19,20].

Currently, there are several approaches to breath-triggered drug release:

For example, a breath-actuated pMDI (pressurized metered-dose inhaler), such as the Autohaler (3M Drug Delivery Systems, St. Paul, MN, USA) or the Tempo inhaler (formerly MAP Pharmaceuticals, Mountain View, CA, USA, now Allergan, Irvine, CA, USA), can be integrated into the ventilation circuit in combination with a spacer. In this case, however, the drug is only released as a single dose via active actuation of the pMDI, by the patient himself or by a third party [21]. Aradigm’s AERx Pulmonary Drug Delivery System (Hayward, CA, USA) follows a similar strategy. This system also releases a single dose, but only if the patient inhales at a flow rate within a pre-defined range [22,23].

Alternatively, an aerosol generator can be coupled to the ventilation circuit by means of an adapter, for example a T-connector [24]. Triggering is achieved via the detection of a pressure change in the ventilation tube, whereupon aerosol production is activated or deactivated; this method is implemented, e.g., in the Aerogen device (Aerogen Ltd., Galway, Ireland) with an integrated control module (Synchro-Neb) [25].

A controlled drug release can also be implemented by means of a pressure measurement at the mouthpiece and an associated evaluation algorithm that considers an average value of the last three breath cycles. A commercial device using this approach is the I-Neb AAD (Philips Respironics, Murrysville, PA, USA). This system releases the pharmaceutical aerosol on the basis of a moving average, which means that the aerosol cannot be released optimally in the event of an aperiodic breathing behavior [26].

In addition, there are pre-calibrated systems, such as the AKITA JET (Vectura Group plc, Chippenham, UK). For such systems, pulmonary parameters must be determined beforehand. The identified parameters are programmed into the system in order to guarantee an optimal aerosol delivery. The system then provides a constant inhalation air flow, delivered by an integrated pump, and starts aerosol production when the patient begins to inhale. As soon as a certain aerosol inhalation volume has been achieved, aerosol production is stopped while the pump continues to provide an inhalation air flow until a defined gas volume is reached. This has the advantage that a particularly large amount of aerosol is deposited in the deeper airways [27].

Moreover, the Fraunhofer Institute for Toxicology and Experimental Medicine (Hannover, Germany) developed a system for the specific requirements of preterm neonates, which manages the breath-triggered drug delivery via a miniaturized valve [28]. This aerosol valve contains an elastomeric membrane that opens and closes symmetrically in less than 25 ms [29]. Due to its miniaturized size, the valve can be integrated directly into a patient interface and is controlled by the detected respiratory signals.

As previously mentioned, breath-triggered drug release devices require breath detection systems that are able to identify the onset of inhalation with high precision and provide appropriate trigger signals. Such systems for breathing detection can be divided into contact-based (either invasive or non-invasive) and non-contact-based (non-invasive) devices [30,31].

There are numerous contact-based methods for measuring respiratory parameters. According to the literature, the best-known contact-based systems are transthoracic impedance measurement, inductive plethysmography, the measurement of abdominal expansion using respiratory belts or the neurally adjusted ventilatory assist method (NAVA) [32,33,34,35]. Transthoracic impedance measurement is a method to derive the respiratory rate by measuring the impedance changes of the chest wall during respiration and is widely used for neonatal respiratory monitoring [36,37]. Respiratory inductive plethysmography, requiring two coils placed in the abdominal and the chest region, respectively, detects changes in self-inductance due to the cross-sectional change of the abdomen during breathing [38,39,40]. The change in inductance is proportional to the lung volume and is successfully used for the detection of respiratory movements in preterm neonates [34,41,42]. Respiratory belts, such as the Graseby capsule (Smiths Medical, Minneapolis, MN, USA) or strain gauges, register the respiratory movement of the abdominal wall by means of pressure sensors, impedance sensors or piezoelectric sensors, which are usually integrated in a single point of measurement on the belt [43,44].

In general, all these systems have a lack of operational reliability because the quality of the measurement strongly depends on the correct placement of the sensor. The applied sensor can cause irritation and damage of the skin or tissue, or, as with NAVA, the placement of the sensor is invasive [32,43,44,45,46]. However, sensor arrays on flexible [47] and even stretchable [48] foils were developed that can be attached to the skin, yielding a higher tolerance to positioning uncertainties. The reliability of these systems is increased by incorporating a high number of individual sensors. The changing shape of the foil can be reconstructed by sophisticated algorithms [49]. Using this stretchable foil, trigger signals were also generated in experiments with neonate models, demonstrating the future potential use to detect inspiration of preterm neonates [50].

Contact-less systems offer some advantages, especially for long-term application, as they do not have to be in permanent and direct contact with the patient. This avoids a stressful situation and pain, which may lead to an increase in the respiratory rate [31]. The best-known contact-less systems for respiratory parameter monitoring are based on flow or pressure sensors at the patient interface [36,51]. However, due to the large technical dead space volume and the susceptibility to aerosol, these sensors are not suitable for monitoring the breathing of (preterm) neonates. In addition, the sensors only function when the patient is intubated and invasively ventilated [52,53]. Therefore, these systems cannot be used for non-invasive ventilation.

Contact-less systems include optical (time-of-flight cameras [54,55], stereo triangulation [56,57] and structured light methods [58,59,60,61,62]), radar [63,64,65,66,67,68], microwave [69,70,71], thermal [60,72,73,74] and laser [75,76] technologies. However, these systems have hardly been clinically tested in preterm neonates. A more suitable respiration monitoring system for (preterm) neonates would be highly desirable. The development and regulatory approval of a breathing detection system for the vulnerable patient group of preterm neonates requires extensive functional tests of the system [77].

An essential challenge in the development of new respiratory sensors, especially contact-based, is that they can only be tested on (preterm) neonates with great effort (including the submission of ethics applications).

For example, a sensor patch for the contact-based registration of a neonate’s breathing cycle was recently developed by Koch et al. [47]. So far, this sensor array foil was tested for mechanical functionality by means of bending experiments. Investigations were also carried out on a ventilated, simple, non-physiological preterm neonate demonstrator in comparison with the measured ventilation flow. However, due to the aforementioned hurdles, no investigations have yet been carried out on preterm neonates in a clinical study. A physiological phantom of the neonatal abdomen enabling simulation of neonatal respiratory patterns would therefore be very useful for rapid, efficient testing and validation of surface-based respiratory sensors. Ethical hurdles would not have to be overcome, and costs could be saved.

To develop such a model, abdominal movement data of preterm neonates are required. Time-of-flight (ToF) cameras are capable of recording depth maps in real time by detecting the phase shift between illumination and reflection and converting it into a distance value [78,79,80]. Furthermore, ToF cameras offer a high spatial resolution and high frame rates and are readily available at a relatively low cost. Therefore, they are well suited for the purpose described above.

It is thus the purpose of the present contribution to introduce and clinically evaluate an optical, non-invasive measurement system enabling the spatial reconstruction of the abdominal movement of a preterm neonate over the course of the respiratory cycle. The obtained 3D recordings, provided as a data set together with this manuscript, represent a valuable starting point for the development of a physiological model of a neonate’s abdominal area which could then be used to circumvent the obstacles described above. This would be an important step towards a clinically available, breath-triggered aerosolization device for the treatment of preterm neonates.

In the following, we demonstrate the feasibility of the derivation of a preterm neonate’s respiratory parameters by recording their abdominal movement using a ToF camera.

## 2. Materials and Methods

According to the regulations for conducting a clinical study at Hannover Medical School, an ethics application was submitted, and the conduct of the study was approved (approval ID: 8584_BO_S_2019).

**Figure 1:**
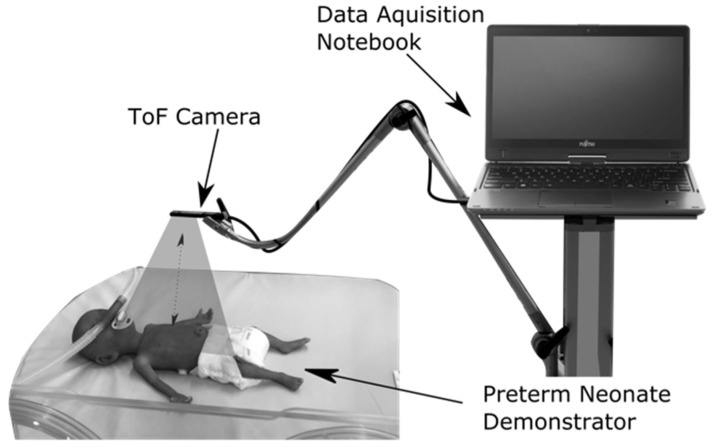
Measurement setup for clinical data recording, exemplified by a preterm neonate demonstrator (NENASim Preemie, Medical-X, Rotterdam, The Netherlands).

We used the CamBoard pico flexx (pmdtechnologies AG, Siegen, Germany) ToF camera to record the abdominal movement data. This ToF camera contains a vertical-cavity surface-emitting laser with a wavelength of 850 nm; it is classified as laser class 1 and is therefore eye-safe [81]. At an object distance of 0.1 m to 1 m and a frame rate of 45 fps, the depth resolution of the camera is ≤2% of the object distance [82]. Preliminary tests showed an optimum distance of 0.2 m between the target region and the camera. For the clinical study, the ToF camera was thus positioned at a distance of 0.2 m above the abdomen of the preterm neonate using a support arm system (Vario Lock, W. Krömker GmbH, Bückeburg, Germany) as shown in Figure 1. The recorded abdominal movement data were transferred to a data acquisition notebook.

In the clinical study, five frame sequences at a duration of 90 s each were acquired with each of five preterm neonates (a total of 25 sequences) at a frame rate of 45 fps (see Table 1 for the clinical parameters of the included neonates).

Python (version 3.8) and the main packages NumPy, SciPy, Pandas and Matplotlib were used for data pre-processing, analysis and evaluation. The initial pre-processing was done in two steps:

First, irrelevant image sections were removed by segmentation. In this process, the center of the ROI was manually selected, and irrelevant sections were removed by means of a cut-off threshold based on the distance to the camera.

Second, faulty pixels and artifacts were removed using a noise reduction method. This method employed a 3D median filter that included a 3×3 pixel neighborhood in the image and five consecutive frames (3×3×5 filter mask). By comparing the standard deviation of all pixels in the ROI over all time points, outliers were automatically removed.

The results of this procedure are pre-processed depth image files that can then be further analyzed and evaluated.

## 3. Results and Discussion

**Figure 2:**
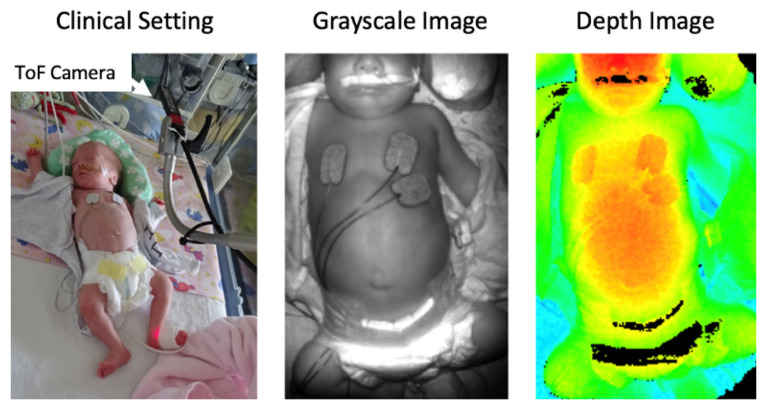
Representation of the measurement setup (**left**) as well as an exemplary 8-bit grayscale (**center**) and 24-bit color-coded (lowest distance to camera: red, largest distance: blue) depth image (**right**) recorded by the ToF system.

Figure 2 shows the placement of the ToF camera above a preterm neonate and the resulting non-pre-processed grayscale and color-coded depth image. The selected ToF camera is particularly suitable for the clinical environment due to its small overall size compared to other systems with similar specifications, such as the Microsoft Kinect 2.0 [83,84] (Microsoft Corp., Redmond, WA, USA), the BlasterX Senz3D [85,86] (Creative Labs (Europe) Ltd., Dublin, Ireland) or the Argos3D-P100 [87] (BECOM Electronics GmbH, Hochstraß, Austria). While the Kinect has a better spatial resolution and a better signal-to-noise-ratio, the ToF camera we used offered a higher frame rate and a better depth resolution, especially at a distance of less than 0.3 m [88,89]. A high frame rate and a high depth resolution are necessary for the accurate detection of breathing movements of preterm neonates because of their high respiratory rate on the one hand and the low amplitude of their abdominal movement in the vertical direction on the other hand. Furthermore, the pico flexx device, at a price of approximately $390, belongs to the low-cost depth cameras, which allows setting up a measuring system for breathing detection economically [89].

**Figure 3:**
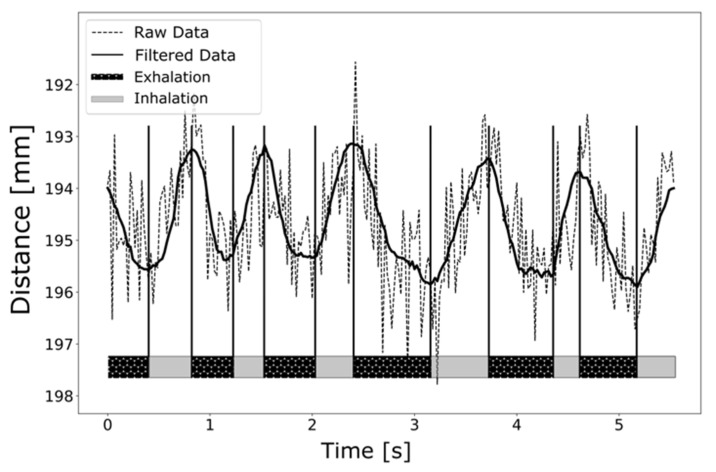
Respiration curve of a preterm neonate, recorded with a pico flexx ToF camera.

The grayscale image contains 256 intensity levels. Highly reflective areas are displayed whitish, and areas of low reflections are shown darker. The depth image was processed with a color map, so that the intensity spectrum contains the entire color scale of human vision from blue (largest distance to the camera) to red (lowest distance to the camera). The highly reflective zones, which are displayed with high intensity in the grayscale image, appear as black artifacts in the depth image, as no valid depth measurement could be performed in these areas. By observing consecutive depth images during the abdominal movement, characteristic distance changes over time, and thus respiratory patterns, can be visualized (see Figure 3).

**Figure 4:**
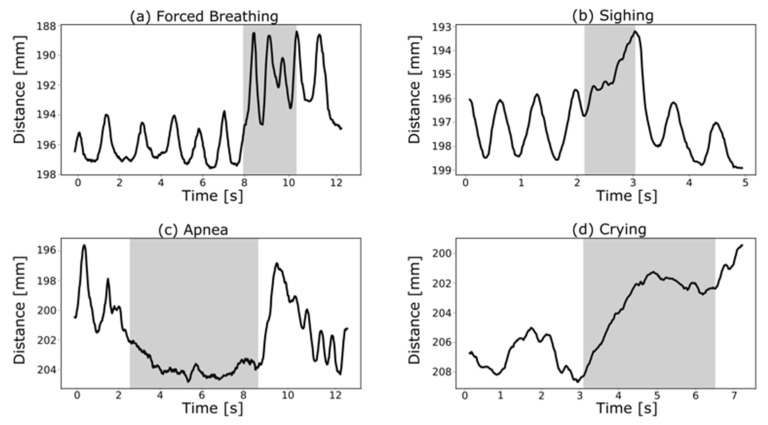
Characteristic breathing patterns, such as forced breathing (**a**), sighing (**b**), apnea (**c**) and crying (**d**), extracted from the clinically acquired ToF recordings.

After filtering the raw data as outlined in Section 2, the temporal evolution of the abdominal movements can be detected very well. The displayed local maxima (maximum abdomen-camera distance) represent the transition from exhalation to inhalation, whereas the local minima (minimum abdomen-camera distance) represent the transition from inhalation to exhalation. The respiratory parameters derived from Figure 3 are 60 breaths/min and an I:E of 1:2.3, which correspond very well with values mentioned in the literature [2,90,91,92]. The average abdominal movement in the vertical direction between maximum inspiration and maximum expiration was (2.3 ± 0.2) mm. Different breathing patterns can be reconstructed from the abdominal movement data (see Figure 4).

After applying a median filter for noise reduction, however, a slight temporal offset could be detected. According to Haju et al., this temporal offset is caused by a phase shift of $\frac{N-1}{2}=2$ frames [93] (N: window size of median filter).

The extracted breathing patterns shown in Figure 4 correspond very well with those described by te Pas et al., e.g., the decrease of the I:E ratio to 1:1 during forced breathing (see Figure 4a) [91]. Besides, spontaneously breathing newborns represent very irregular respiratory patterns such as periodic breathing [94], apneas [95] and sighs [96] in order to recruit lung volume. Figure 4b depicts a sigh, followed by an apneic pause and subsequently a slower respiratory rate, whereas Figure 4c shows an apneic phase of about 5 s. Crying is defined by a strong inhalation followed by an interrupted exhalation [97], which can be observed in Figure 4d.

Another common breathing pattern seen in preterm neonates is paradoxical breathing, in which the chest pulls inward on inhalation and outward on exhalation. This often occurs when the thorax is unstable. Because the bony thorax of preterm neonates is very elastic and gives way during forced breaths, this breathing pattern is commonly seen [98]. However, as (preterm) neonates are obligate abdominal breathers, the present study is focused on recording abdominal movements. We are confident that by focusing on abdominal movements, our system allows for the reliable detection of the inspiratory phase, even if a paradoxical thoracic movement should occur.

Further, we can confirm that the proposed method is not suitable for the detection of obstructive events, as it does not evaluate gas flow. However, we believe that this is of limited clinical relevance because apneic episodes (central, obstructive, as well as mixed) occur regularly in preterm neonates, leading to temporary gas flow interruption.

**Figure 5:**
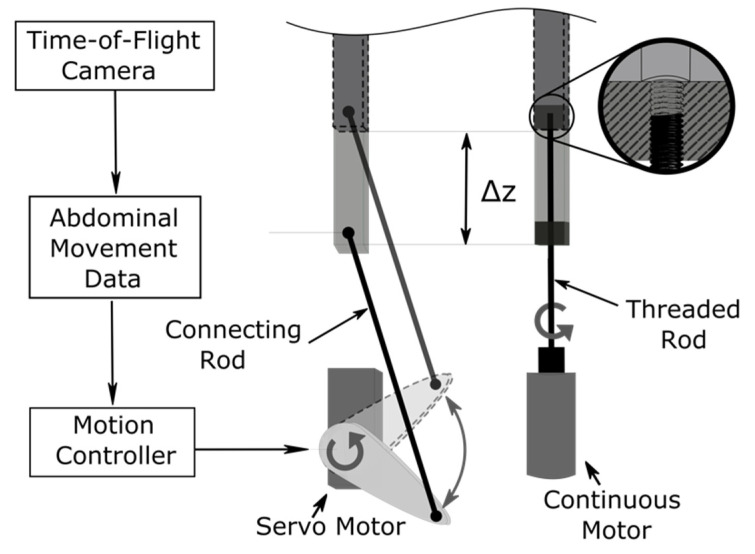
Example of shifting individual pins by ∆z by means of a servo motor (**left**) and by means of a continuous motor (**right**) to simulate abdominal movement [101].

Using the recorded movement data, it is now possible to develop a physical abdominal model, for example on the basis of an actuated pin matrix [99,100]. These are also known as Tangible User Interfaces. The concept consists of a matrix of pins, each controlled individually or as a group by an actuator. Figure 5 (left) shows a servo motor driving a connecting rod that is attached to an individual pin. As a result, this setup requires slightly more space than the setup on the right in Figure 5, in which a continuous motor drives a threaded rod, thereby changing its height by ∆z.

By controlling the individual pins independently, three-dimensional shapes can be represented, and movements can also be reproduced in a desired form. Thus, using the abdominal movement data generated with the ToF camera, breathing patterns such as crying and sighing can be exactly simulated. This allows the initial testing of newly-developed (contact-based) sensors on such a model before being made available for clinical purposes. After validation, such sensor systems can be used for real-time breath detection, making it suitable for the application of breath-triggered drug release for (preterm) neonates [28,29,102].

Further, we believe that our depth-sensing approach may be advantageous in estimating the functional residual capacity (FRC), as it provides a three-dimensional representation of the neonatal abdomen. However, our data processing algorithm would have to adapted to obtain an estimation of the temporal evolution of the abdominal volume. The Time-of-Flight sequences of our study may serve as a data set to develop and evaluate future algorithms for FRC estimation.

## 4. Conclusions

We showed that recording respiratory parameters, as well as respiratory monitoring, is possible without any patient contact using a ToF camera in preterm neonates. Therefore, the proposed optical measuring system is deemed suitable for a wide range of neonatal applications, for example, general patient monitoring or, in particular, breathing detection. In principle, the contact-less recording by a ToF camera offers strong advantages over a contact-based method: The preterm neonate is neither affected by the ToF camera, nor does this measurement technique require patient interaction. Therefore, such a system is suitable as a source of a trigger signal for the application of breath-triggered drug release in (preterm) neonates.

Furthermore, the acquired abdominal movement data are also suitable for the development and control of an abdominal movement model. Sensors for the detection of abdominal movements could first be tested on such a model before being used in a clinical setting.

## 5. Outlook

The present work constitutes the first step towards the efficient and ethical evaluation of novel breath phase sensors for inspiration-triggered drug delivery systems using an actuated phantom of the patient’s abdominal area. The spatial movement data acquired in the present contribution could be mapped onto a pin matrix, as described in Section 3. The shape of the surface formed by the individual pins could be adapted to the surface topography of the abdomen, identified during the acquisition of the abdominal motion, to obtain an even higher accuracy of the simulated breathing activity.

In the future, a multi-body system, inspired by the anatomy of the neonatal abdomen, could be developed and used to validate the physiology of the phantom’s motions over a breathing cycle.

In conclusion, in the progress in the fields of high-precision breath cycle detection, controlled aerosol delivery and drug discovery will contribute to the establishment of more effective and targeted strategies for the treatment of preterm neonates by inhalation therapy.

## Figures and Tables

**Table 1:** Clinical parameters of neonates included in this study.

ID	Gender	Gestational Age at Birth [Weeks]	Postmenstrual Age at Recording [Weeks]	Weight at Birth [g]	Weight at Recording [g]	Therapy Form
1	Male	25	32 1/7	800	1580	High-flow nasal cannula
2	Male	32 1/7	33 1/7	755	720	Spontaneous breathing
3	Male	27 3/7	33 1/7	585	1245	High-flow nasal cannula
4	Female	27 3/7	33 1/7	930	1920	Spontaneous breathing
5	Male	27 3/7	33 1/7	860	1550	Spontaneous breathing

## Data Availability

The complete set of Time-of-Flight sequences which we recorded in our work will be uploaded and made available in the publicly available research data repository of the Fraunhofer Society (Fordatis) (https://fordatis.fraunhofer.de, accessed on 22 April 2021).

## References

[1] Fink J.B. (2004). Aerosol delivery to ventilated infant and pediatric patients. Respir. Care.

[2] Walsh B.K., Czervinske M.P., DiBlasi R.M. (2010). Perinatal and Pediatric Respiratory Care.

[3] Cole C.H. (2000). Special problems in aerosol delivery: Neonatal and pediatric considerations. Respir. Care.

[4] Dhand R. (2008). Aerosol delivery during mechanical ventilation: From basic techniques to new devices. J. Aerosol Med. Pulm. Drug Deliv..

[5] Turpeinen M., Nikander K. (2001). Nebulization of a suspension of budesonide and a solution of terbutaline into a neonatal ventilator circuit. Respir. Care.

[6] Longest P.W., Azimi M., Hindle M. (2014). Optimal Delivery of Aerosols to Infants During Mechanical Ventilation. J. Aerosol Med. Pulm. Drug Deliv..

[7] Chua H.L., Collis G.G., Newbury A.M., Chan K., Bower G.D., Sly P.D., Le Souef P.N. (1994). The influence of age on aerosol deposition in children with cystic fibrosis. Eur. Respir. J..

[8] Mallol J., Rattray S., Walker G., Cook D., Robertson C.F. (1996). Aerosol deposition in infants with cystic fibrosis. Pediatr. Pulmonol..

[9] Fok T.F., Monkman S., Dolovich M., Gray S., Coates G., Paes B., Rashid F., Newhouse M., Kirpalani H. (1996). Efficiency of aerosol medication delivery from a metered dose inhaler versus jet nebulizer in infants with bronchopulmonary dysplasia. Pediatr. Pulmonol..

[10] Salmon B., Wilson N.M., Silverman M. (1990). How much aerosol reaches the lungs of wheezy infants and toddlers?. Arch. Dis. Child..

[11] Köhler E., Jilg G., Avenarius S., Jorch G. (2008). Lung deposition after inhalation with various nebulisers in preterm infants. Arch. Dis. Child. Fetal Neonatal Ed..

[12] Pelkonen A.S., Nikander K., Turpeinen M. (1997). Jet nebulization of budesonide suspension into a neonatal ventilator circuit: Synchronized versus continuous nebulizer flow. Pediatr. Pulmonol..

[13] Huang C.-W., Pei C., Huang C.-H. (2011). Respiratory deposition model of an inhaled aerosol bolus. Comput. Methods Biomech. Biomed. Engin..

[14] Miller D.D., Amin M.M., Palmer L.B., Shah A.R., Smaldone G.C. (2003). Aerosol delivery and modern mechanical ventilation: In vitro/in vivo evaluation. Am. J. Respir. Crit. Care Med..

[15] Zeman K.L., Wu J., Bennett W.D. (2010). Targeting aerosolized drugs to the conducting airways using very large particles and extremely slow inhalations. J. Aerosol Med. Pulm. Drug Deliv..

[16] Fischer A., Stegemann J., Scheuch G., Siekmeier R. (2009). Novel devices for individualized controlled inhalation can optimize aerosol therapy in efficacy, patient care and power of clinical trials. Eur. J. Med Res..

[17] Scheuch G. (1994). Particle recovery from human conducting airways after shallow aerosol bolus inhalation. J. Aerosol Sci..

[18] Scheuch G., Siekmeier R. (2007). Novel approaches to enhance pulmonary delivery of proteins and peptides. J. Physiol. Pharm..

[19] Martin A.R. (2015). Deposition of Aerosols in the Lungs: Regional Deposition–Targeting. ISAM Textbook of Aerosol Medicine.

[20] Heyder J. (2004). Deposition of inhaled particles in the human respiratory tract and consequences for regional targeting in respiratory drug delivery. Proc. Am. Thorac. Soc..

[21] Newman S.P. (2005). Principles of metered-dose inhaler design. Respir. Care.

[22] Cipolla D., Johansson E., Rathbone M.J. (2008). AERx^®^Pulmonary Drug Delivery Systems. Modified-Release Drug Delivery Technology.

[23] Cipolla D., Chan H.-K., Schuster J., Farina D. (2010). Personalizing aerosol medicine: Development of delivery systems tailored to the individual. Ther. Deliv..

[24] Dhand R. (2012). Aerosol therapy in patients receiving noninvasive positive pressure ventilation. J. Aerosol Med. Pulm. Drug Deliv..

[25] Michotte J.-B., Staderini E., Le Pennec D., Dugernier J., Rusu R., Roeseler J., Vecellio L., Liistro G., Reychler G. (2016). In Vitro Comparison of a Vibrating Mesh Nebulizer Operating in Inspiratory Synchronized and Continuous Nebulization Modes During Noninvasive Ventilation. J. Aerosol Med. Pulm. Drug Deliv..

[26] Geller D.E., Kesser K.C. (2010). The I-neb Adaptive Aerosol Delivery System enhances delivery of alpha1-antitrypsin with controlled inhalation. J. Aerosol Med. Pulm. Drug Deliv..

[27] Fink J.B., Stapleton K.W. (2015). Nebulizers. ISAM Textbook of Aerosol Medicine.

[28] Pohlmann G., Wiegandt F.C., Iwatschenko P. Fraunhofer-Gesellschaft zur Förderung der Angewandten Forschung e.V.: Respiration-Controlled Application of Aerosol in Powder Form during the Artificial Respiration or Supported Respiration of a Patient. WO/2018/010954 A1, 18 January 2018. https://patents.google.com/patent/WO2018010954A1/en.

[29] Wiegandt F.C., Koch E., Iwatschenko P., Dietzel A., Pohlmann G. (2017). Pre-triggered dry and liquid aerosol release inside the patient interface of preterm neonates. J. Aerosol Med. Pulm. Drug Deliv..

[30] Massaroni C., Nicolò A., Lo Presti D., Sacchetti M., Silvestri S., Schena E. (2019). Contact-Based Methods for Measuring Respiratory Rate. Sensors.

[31] Al-Khalidi F.Q., Saatchi R., Burke D., Elphick H., Tan S. (2011). Respiration rate monitoring methods: A review. Pediatr. Pulmonol..

[32] Stein H., Firestone K. (2014). Application of neurally adjusted ventilatory assist in neonates. Semin. Fetal Neonatal Med..

[33] Stick S.M., Ellis E., LeSouëf P.N., Sly P.D. (1992). Validation of respiratory inductance plethysmography (“Respitrace”) for the measurement of tidal breathing parameters in newborns. Pediatr. Pulmonol..

[34] Locke R., Greenspan J.S., Shaffer T.H., Rubenstein S.D., Wolfson M.R. (1991). Effect of nasal CPAP on thoracoabdominal motion in neonates with respiratory insufficiency. Pediatr. Pulmonol..

[35] Emeriaud G., Eberhard A., Benchetrit G., Debillon T., Baconnier P. (2008). Calibration of respiratory inductance plethysmograph in preterm infants with different respiratory conditions. Pediatr. Pulmonol..

[36] Di Fiore J.M. (2004). Neonatal cardiorespiratory monitoring techniques. Semin. Neonatol..

[37] Kohn S., Waisman D., Pesin J., Faingersh A., Klotzman I.C., Levy C., Hirshberg G., Rotschild A., Landesberg A. (2016). Monitoring the respiratory rate by miniature motion sensors in premature infants: A comparative study. J. Perinatol..

[38] Retory Y., Niedzialkowski P., de Picciotto C., Bonay M., Petitjean M., Cooper C. (2016). New Respiratory Inductive Plethysmography (RIP) Method for Evaluating Ventilatory Adaptation during Mild Physical Activities. PLoS ONE.

[39] Cohn M.A., Rao A.S., Broudy M., Birch S., Watson H., Atkins N., Davis B., Stott F.D., Sackner M.A. (1982). The respiratory inductive plethysmograph: A new non-invasive monitor of respiration. Bull. Eur. Physiopathol. Respir..

[40] Chadha T.S., Watson H., Birch S., Jenouri G.A., Schneider A.W., Cohn M.A., Sackner M.A. (1982). Validation of respiratory inductive plethysmography using different calibration procedures. Am. Rev. Respir. Dis..

[41] Warren R.H., Horan S.M., Robertson P.K. (1997). Chest wall motion in preterm infants using respiratory inductive plethysmography. Eur. Respir. J..

[42] Poole K.A., Thompson J.R., Hallinan H.M., Beardsmore C.S. (2000). Respiratory inductance plethysmography in healthy infants: A comparison of three calibration methods. Eur. Respir. J..

[43] De Waal C.G., Kraaijenga J.V., Hutten G.J., de Jongh F.H., van Kaam A.H. (2017). Breath detection by transcutaneous electromyography of the diaphragm and the Graseby capsule in preterm infants. Pediatr. Pulmonol..

[44] Stern D.J., Weisner M.D., Courtney S.E. (2014). Synchronized neonatal non-invasive ventilation-a pilot study: The graseby capsule with bi-level NCPAP. Pediatr. Pulmonol..

[45] Sinderby C., Navalesi P., Beck J., Skrobik Y., Comtois N., Friberg S., Gottfried S.B., Lindström L. (1999). Neural control of mechanical ventilation in respiratory failure. Nat. Med..

[46] Baird T.M., Neuman M.R. (1991). Effect of infant position on breath amplitude measured by transthoracic impedance and strain gauges. Pediatr. Pulmonol..

[47] Koch E., Dietzel A. (2016). Skin attachable flexible sensor array for respiratory monitoring. Sens. Actuators A Phys..

[48] Koch E., Dietzel A. Stretchable sensor array for respiratory monitoring. Proceedings of the 2017 19th International Conference on Solid-State Sensors, Actuators and Microsystems (TRANSDUCERS).

[49] Koch E., Dietzel A. (2017). Surface reconstruction by means of a flexible sensor array. Publ. Inst. Microtechnol. IMT.

[50] Koch E., Dietzel A. (2018). Respiratory trigger signal generation by means of a stretchable sensor array. Sensors Actuator A Phys..

[51] Anderson J.V., Martin R.J., Lough M.D., Martinez A. (1982). An improved nasal mask pneumotachometer for measuring ventilation in neonates. J. Appl. Physiol. Respir. Environ. Exerc. Physiol..

[52] Greenough A., Milner A. (1997). Patient-triggered ventilation using flow or pressure sensors. Semin. Neonatol..

[53] Mazela J., Polin R.A. (2011). Aerosol delivery to ventilated newborn infants: Historical challenges and new directions. Eur. J. Pediatr..

[54] Penne J., Schaller C., Hornegger J., Kuwert T. (2008). Robust real-time 3D respiratory motion detection using time-of-flight cameras. Int. J. Comput. Assist. Radiol. Surg..

[55] Placht S., Stancanello J., Angelopoulou E., Schaller C., Balda M. (2012). Fast time-of-flight camera based surface registration for radiotherapy patient positioning. Am. Assoc. Phys. Med..

[56] Spinczyk D., Karwan A., Copik M. (2014). Methods for abdominal respiratory motion tracking. Comput. Aided Surg..

[57] Bartula M., Tigges T., Muehlsteff J. Camera-based system for contactless monitoring of respiration. Proceedings of the 35th Annual International Conference of the IEEE Engineering in Medicine and Biology Society (EMBC).

[58] Cattani L., Alinovi D., Ferrari G., Raheli R., Pavlidis E., Spagnoli C., Pisani F. (2017). Monitoring infants by automatic video processing: A unified approach to motion analysis. Comput. Biol. Med..

[59] Lim S.H., Golkar E., Abd Rahni A.A. Respiratory motion tracking using the kinect camera. Proceedings of the IEEE Conference on Biomedical Engineering and Sciences (IECBES).

[60] Procházka A., Charvátová H., Vyšata O., Kopal J., Chambers J. (2017). Breathing Analysis Using Thermal and Depth Imaging Camera Video Records. Sensors.

[61] Sharp C., Soleimani V., Hannuna S., Camplani M., Damen D., Viner J., Mirmehdi M., Dodd J.W. (2017). Toward Respiratory Assessment Using Depth Measurements from a Time-of-Flight Sensor. Front. Physiol..

[62] Gleichauf J., Niebler C., Koelpin A. Automatic non-contact monitoring of the respiratory rate of neonates using a structured light camera. Proceedings of the 2020 42nd Annual International Conference of the IEEE Engineering in Medicine & Biology Society (EMBC).

[63] Nijsure Y., Tay W.P., Gunawan E., Wen F., Yang Z., Guan Y.L., Chua A.P. (2013). An impulse radio ultrawideband system for contactless noninvasive respiratory monitoring. IEEE Trans. Biomed. Eng..

[64] Liang X., Wang Y., Wu S., Gulliver T.A. (2018). Experimental Study of Wireless Monitoring of Human Respiratory Movements Using UWB Impulse Radar Systems. Sensors.

[65] Lv H., Jiao T., Zhang Y., Liang F., Qi F., Wang J. (2018). A Novel Method for Breath Detection via Stepped-Frequency Continuous Wave Ultra-Wideband (SFCW UWB) Radars Based on Operational Bandwidth Segmentation. Sensors.

[66] Greneker E.F. Radar sensing of heartbeat and respiration at a distance with applications of the technology. Proceedings of the Radar Systems (RADAR 97).

[67] Schreurs D., Mercuri M. Contactless medical sensing. Proceedings of the 2015 IEEE MTT-S International Microwave Symposium (IMS2015).

[68] Zito D., Pepe D. Monitoring respiratory pattern in adult and infant via contactless detection of thorax and abdomen movements through SoC UWB pulse radar sensor. Proceedings of the Biomedical Wireless Technologies, Networks, and Sensing Systems (BioWireleSS), 2014 IEEE Topical Conference on IEEE.

[69] Dei D., Grazzini G., Luzi G., Pieraccini M., Atzeni C., Boncinelli S., Camiciottoli G., Castellani W., Marsili M., Lo Dico J. (2009). Non-contact detection of breathing using a microwave sensor. Sensors.

[70] Franks C.I., Brown B.H., Johnston D.M. (1976). Contactless respiration monitoring of infants. Med. Biol. Engng..

[71] Gu C. (2016). Short-Range Noncontact Sensors for Healthcare and Other Emerging Applications: A Review. Sensors.

[72] Lorato I., Stuijk S., Meftah M., Kommers D., Andriessen P., van Pul C., de Haan G. (2020). Multi-camera infrared thermography for infant respiration monitoring. Biomed. Opt. Express.

[73] Johnson M.L., Price P.A., Jovanov E. (2007). A new method for the quantification of breathing. Annu. Int. Conf. IEEE Eng. Med. Biol. Soc..

[74] Lorato I., Bakkes T., Stuijk S., Meftah M., Haan G. de. (2019). Unobtrusive Respiratory Flow Monitoring Using a Thermopile Array: A Feasibility Study. Appl. Sci..

[75] Scalise L., Ercoli I., Marchionni P., Tomasini E.P. Measurement of respiration rate in preterm infants by laser Doppler vibrometry. Proceedings of the 2011 IEEE International Symposium on Medical Measurements and Applications (MeMeA).

[76] Wiegandt F.C., Froriep U.P., Doll T., Dietzel A., Pohlmann G. (2020). Novel Test Bench for Inhaler Characterization Including Real-Time Determination of Output, Output Rate, and Liquid Water Content of Delivered Aerosols. J. Aerosol Med. Pulm. Drug Deliv..

[77] (2017). Regulation (EU) 2017/745 of the European Parliament and of the Council of 5 April 2017 on medical devices, amending Directive 2001/83/EC, Regulation (EC) No 178/2002 and Regulation (EC) No 1223/2009 and repealing Council Directives 90/385/EEC and 93/42/EEC (Text with EEA relevance.): MDR. Official Journal of the European Union.

[78] Hansard M., Lee S., Choi O., Horaud R. (2013). Time-of-Flight Cameras.

[79] Schaller C., Penne J., Hornegger J. (2008). Time-of-flight sensor for respiratory motion gating. Med. Phys..

[80] Müller K., Schaller C., Penne J., Hornegger J., Brauer W., Meinzer H.-P., Deserno T.M., Handels H., Tolxdorff T. (2009). Surface-Based Respiratory Motion Classification and Verification. Bildverarbeitung für Die Medizin 2009: Algorithmen-Systeme-Anwendungen.

[81] International Organization for Standardization Safety of Laser Products-Part. 1: Equipment Classification and Requirements (IEC 60825-1:2014), 2014, 13.110 Safety of Machinery, 31.260 Optoelectronics. Laser Equipment (IEC 60825-1:2014). https://webstore.iec.ch/publication/3587.

[82] PMD Technologies AG Development Kit Brief CamBoard Pico Flexx. https://pmdtec.com/picofamily/wp-content/uploads/2018/03/PMD_DevKit_Brief_CB_pico_flexx_CE_V0218-1.pdf.

[83] Gambi E., Agostinelli A., Belli A., Burattini L., Cippitelli E., Fioretti S., Pierleoni P., Ricciuti M., Sbrollini A., Spinsante S. (2017). Heart Rate Detection Using Microsoft Kinect: Validation and Comparison to Wearable Devices. Sensors.

[84] Al-Naji A., Gibson K., Lee S.-H., Chahl J. (2017). Real Time Apnoea Monitoring of Children Using the Microsoft Kinect Sensor: A Pilot Study. Sensors.

[85] Siena F.L., Byrom B., Watts P., Breedon P., Blashki K., Xiao Y. (2018). Assessment of Facial Tracking For Use In Clinical Outcome & Applications. Proceedings of the International Conferences Interfaces and Human Computer Interaction 2018, Game and Entertainment Technologies 2018 and Computer Graphics, Visualization, Computer Vision and Image Processing 2018.

[86] Przybyło J. (2019). Continuous Distant Measurement of the User’s Heart Rate in Human-Computer Interaction Applications. Sensors.

[87] Nicolai P., Raczkowsky J., Wörn H., Filipe J., Madani K., Gusikhin O., Sasiadek J. (2015). Continuous Pre-Calculation of Human Tracking with Time-delayed Ground-truth-A Hybrid Approach to Minimizing Tracking Latency by Combination of Different 3D Cameras. Proceedings of the 2015 12th International Conference on Informatics in Control, Automation and Robotics (ICINCO).

[88] Pasinetti S., Hassan M.M., Eberhardt J., Lancini M., Docchio F., Sansoni G. (2019). Performance Analysis of the PMD Camboard Picoflexx Time-of-Flight Camera for Markerless Motion Capture Applications. IEEE Trans. Instrum. Meas..

[89] Condotta I.C., Brown-Brandl T.M., Pitla S.K., Stinn J.P., Silva-Miranda K.O. (2020). Evaluation of low-cost depth cameras for agricultural applications. Comput. Electron. Agric..

[90] te Pas A.B., Davis P.G., Kamlin C.O.F., Dawson J., O’Donnell C.P.F., Morley C.J. (2008). Spontaneous breathing patterns of very preterm infants treated with continuous positive airway pressure at birth. Pediatr. Res..

[91] te Pas A.B., Wong C., Kamlin C.O.F., Dawson J.A., Morley C.J., Davis P.G. (2009). Breathing patterns in preterm and term infants immediately after birth. Pediatr. Res..

[92] von Mutius E., Gappa M., Eber E., Frey U. (2013). Pädiatrische Pneumologie.

[93] Harju P.T., Ovaska S.J., Valimaki V. Delayless signal smoothing using a median and predictive filter hybrid. Proceedings of the Third International Conference on Signal Processing (ICSP’96).

[94] Abu-Shaweesh J.M. (2004). Maturation of respiratory reflex responses in the fetus and neonate. Semin. Neonatol..

[95] Finer N.N., Higgins R., Kattwinkel J., Martin R.J. (2006). Summary proceedings from the apnea-of-prematurity group. Pediatrics.

[96] Poets C.F., Rau G.A., Neuber K., Gappa M., Seidenberg J. (1997). Determinants of lung volume in spontaneously breathing preterm infants. Am. J. Respir. Crit. Care Med..

[97] Halverson H.M. (1941). Variations in Pulse and Respiration during Different Phases of Infant Behavior. Pedagog. Semin. J. Genet. Psychol..

[98] Heldt G.P., McIlroy M.B. (1987). Dynamics of chest wall in preterm infants. J. Appl. Physiol..

[99] Siu A.F., Gonzalez E.J., Yuan S., Ginsberg J., Zhao A., Follmer S. shapeShift: A Mobile Tabletop Shape Display for Tangible and Haptic Interaction. Proceedings of the UIST ’17: The 30th Annual ACM Symposium on User Interface Software and Technology.

[100] Follmer S., Leithinger D., Olwal A., Hogge A., Ishii H., Izadi S., Quigley A., Poupyrev I., Igarashi T. (2013). inFORM: Dynamic Physical Affordances and Constraints through Shape and Object Actuation. Proceedings of the 26th annual ACM symposium on User Interface Software and Technology-UIST ‘13.

[101] Biegger D. (2019). Entwicklung Eines Modells zur Simulation der Physiologischen Abdominalbewegung von Frühgeborenen Während der Atmung zur Charakterisierung Eines Sensorpatches. Master Thesis.

[102] Wiegandt F.C., Froriep U.P., Müller F., Doll T., Dietzel A., Pohlmann G. (2021). Breath-Triggered Drug Release System for Preterm Neonates. Pharmaceutics.

